# Supplementary material for: Dietary Choline Mitigates High-Fat Diet-Impaired Chylomicrons Assembly via UPRer Modulated by perk DNA Methylation
Source: Cells. 2022 Nov 30;11(23):3848. doi: 10.3390/cells11233848 (PMC9741040; doi:10.3390/cells11233848)
Supplement: Supplementary file 1 [file cells-11-03848-s001.zip › cells-2040614-supplementary.pdf]

Table S1. Feed formulation and proximate analysis of experimental diets.

Ingredients (g kg $^{-1}$)	Control	HFD	CH	HFD + CH
Casein	360	360	360	360
Gelatin	80	80	80	80
Fish oil	30	45	30	45
Corn oil	30	45	30	45
Wheat flour	250	250	250	250
Ascorbyl-2-polyphosphate	10	10	10	10
NaCl	10	10	10	10
Ca $(H_2PO_4)_2 \cdot H_2O$	10	10	10	10
Vitamin premix[1]	5	5	5	5
Mineral premix[2]	5	5	5	5
Cellulose	209.5	179.5	208.5	179.5
Choline chloride	0.5	0.5	1.5	1.5
Proximate analysis (%, dry weight)				
Moisture	7.22	7.38	7.29	7.34
Crude protein	39.99	40.86	40.54	40.23
Crude ash	5.37	5.29	5.36	5.38
Crude lipid	10.15	14.61	10.49	14.65
Choline (mg kg^{-1})	563.4	578.9	1650.1	1652.3

[1]Vitamin premix (mg or IU per kg diet): retinylacetate, 10000IU; cholecalciferol, 1000IU; all-rac-a- tocopheryl acetate, 30IU; menadione nicotinamide bisulfite, 7; thiamine hydrochloride, 6; riboflavin, 3; pyridoxine hydrochloride, 12; D-calcium pantothenate, 30; niacin, 50; biotin, 1; folic acid, 6; cyanocobalamine, 0.03.

[2]Mineral mixture (mg per kg diet): $Ca(H_2PO_3)_2 \cdot H_2O$, 1000; $FeSO_4 \cdot 7H_2O$, 40; $ZnSO_4 \cdot 7H_2O$, 40; $MnSO_4 \cdot H_2O$, 40; $CuSO_4 \cdot 5H_2O$, 2; $CaIO_3 \cdot 6H_2O$, 3; Na_2SeO_3, 0.05; $CoSO_4$, 0.05.

Table S2. Effects of dietary high fat and choline supplementation on growth performance and morphological parameters yellow catfish *(Pelteobagrus fulvidraco)* after 10 weeks.

Diet	Control	HFD	CH	HFD+CH	Two-way ANOVA *P*-vaule		
					Fat	Choline	Interaction
**IBW, g/fish**	3.78±0.05	3.78±0.03	3.80±0.06	3.85±0.01	NS	NS	NS
**FBW, g/fish**	31.38±0.91[ab]	35.10±0.14[c]	30.03±0.63[a]	32.00±0.50[b]	<0.001	<0.001	0.034
**WG[2], %**	728.60±0.15[ab]	827.08±.061[c]	690.16±0.10[a]	730.60±0.16[b]	<0.001	<0.001	0.049
**FCR[3]**	1.15±0.03	1.08±0.03	1.17±0.02	1.14±0.01	0.025	NS	NS
**HSI[4], %**	1.37±0.27	1.43±0.17	1.30±0.25	1.40±0.20	NS	0.007	NS
**CF[5], %**	1.70±0.06	1.68±0.30	1.69±0.34	1.72±0.15	NS	NS	NS
**Survival[6], %**	98.66±2.30	98.66±2.30	96.00±4.00	97.33±3.21	NS	NS	NS

[1]Values are means ± SEM; n = 3 tanks (30 fish/tank). Labeled means without a common letter differ, $P < 0.05$ (TWO-factor ANOVA, Duncan's post hoc test).

[2]WG = (FBW-IBW)/IBW×100.

[3]FCR = dry feed fed (g)/wet weight gain (g).

[4]HSI = 100×(liver weight)/(body weight);

[5]CF = 100×(live weight, g)/(body length, cm)^3.

[6]Survival = 100 ×final fish number/initial fish number.

Table S3. Primers used for plasmid construction and RT-qPCR analysis

Gene	Accession No.	Forward primer (5'-3')	Reverse primer (5'-3')
**Bisulfite sequencing for methylation validation**			
perk	KP687344.1	TTTAGGGTTGGGTTATTTAGAAATG	ATCTTCTCTCTCATATCAATCTCATATACA
**Plasmid construction**			
perk	KP687344.1	ctagcgtttaaacttaagcttATGGAGCAAGCAAACCTCTACG	aacgggccctctagactcgagTTAGGCACAGTTACCCATGGC
**RT-qPCR analysis**			
18s	EU047719	GGCGCGCAAATTACCCATT	TCCCGAGATCCAACTACAAGC
β-actin	DQ211096.1	ACCCTGAAGTACCCCATCGA	CAGAGGCATACAGGGACAGC
accα	GU908474.1	GCTTGCGGCGGTTATTACTG	AGCTGCCTCTCCAACCATTC
b2m	AB128864.1	GCACTCGTCTCTTTTGCCCT	TTTCGAAGGCCAGGTCAGTC
dnmt1	MN701649	TCATGTGCGGGAACAACAAC	TGCAGCACATGTAGCAGTTC
dnmt3a	MN701650	TGCAGCACTTCTTTGCCAAC	GACAGGACACGAATGGGTTTTC
dnmt3b	MN701651	ACCTGGAATGAACCGACCAC	ATTCATGGTGACCGGCAGAG
fas	GU433188	TCATCCAGCAGTTCACTGGCATT	TGATTAGGTCCACGGCCACA
6pgd	XM003444904.4	GAAGGGCCTGCTGTTTGTTG	CCCAGTCACAACAAGGCTCT
g6pd	XM005478106	GAGAAGCCCTTTGGTCGTGA	ATCAAAGTACCCTCCACGGC
dgat1	MN701645	AACGAAAGACTGCGCAAGAG	ACCCATGGCTTTGACAAACG
dgat2	MN701646	TTCCGGGAACTTTGACATGC	GGTTGCGCATTTTGGCTTTG
mgat1	GQ266394.1	CCACCGGCCATCTGATCTAC	GTGTCCAGGGGCATCAATGA
mgat2	MG241310.1	TTGTGGTCTTCTCCTCGGTC	ACGACACCCACATCTCCAAT
grp78	FJ436356.1	ATTTGTTCCGCTCCACCATG	AACTCTTTCACCAGCTGCTG
perk	KP687344.1	GGGAAACTGTGGAGGGATGG	TGCAGCCTTGACCACTTTCT

eif2α	JN195739.1	TCGGCCCCAGTCTCATTCTA	ATACACCACTCGCCTCTCCT
ire1α	KY081668.1	TTCTGCGGGAAACGTTTCAC	ACTACGCATGAACCGTTTGG
xbp1	MN701647	CTCCTGAACAGAAGCAGCCA	CTCGAAGTGCTCTGCCATGA
atf6	XM005471382	TCCCCGGATCATCGTATGGA	TCCTGCAGTGACTCCTAACG
apob	KF871430	TCCCCGGATCATCGTATGGA	TCCTGCAGTGACTCCTAACG
apobec1	XM003447923.5	GATCCTCACTACTGCCAGCC	CCTTCGACGATGAGAGAGCC
apoe	XM003450274.5	GACCAGTTCTGGGCCATGAA	GGTGTATGCGTTGCCTACAG
mtp	XM025907253.1	GATGTTGTGGCACCAGGAGA	CATTCTGTCATCGCTGCTGC
sar1b	XM005476433.4	TACTGGTTCACGTGCCTGAG	GCTCGATTGGCAAACAGGAC
cd36	XM019356796.2	GTCCAGCAGATCCGTGAGC	TGCCAGGAACTTGGTCTTGTC
vamp7	XM003439438.5	GCCTCCAGCTAAACTCCAACC	CGCTCTCGCTCGTACACCTC
**siRNA sequences**			
siRNA-*perk*-376		GCGAGCACCGUGCAGUUUATT	UAAACUGCACGGUGCUCGCTT
NC-siRNA		UUCUCCGAACGUGUCACGUTT	ACGUGACACGUUCGGAGAATT

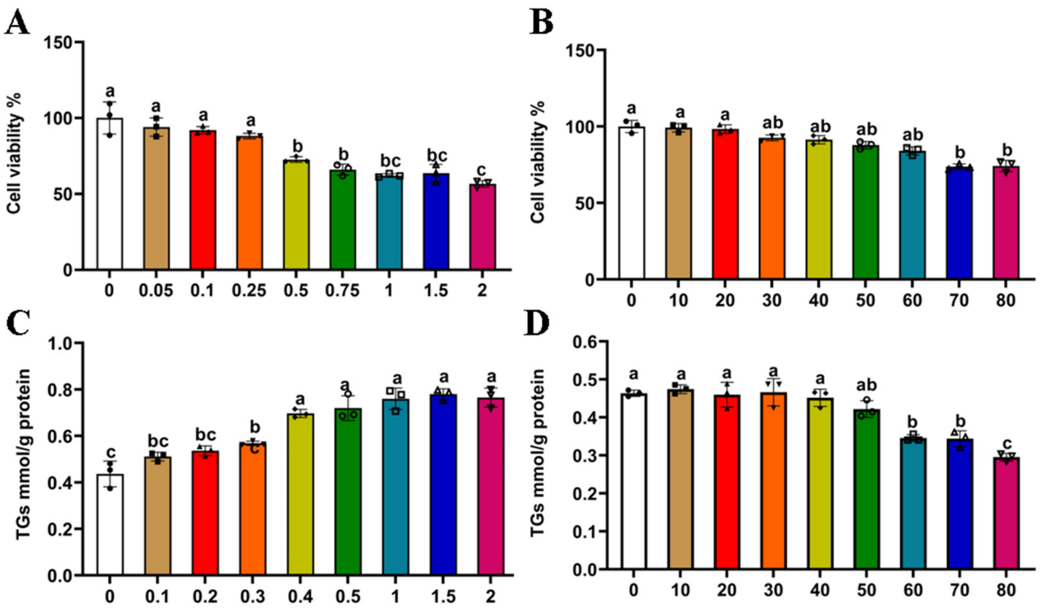

Figure S1. MTT assay and TGs content for the viability of intestinal epithelial cells (IECs) of yellow catfish incubated with FA and choline for 36h. (A-B) MTT assay of FA and choline. (C-D) TG content of FA and choline. Values are means ± SEM, n ≥ 3. Letters denote significance at $P < 0.05$ (One-way ANOVA, Duncan post hoc test).

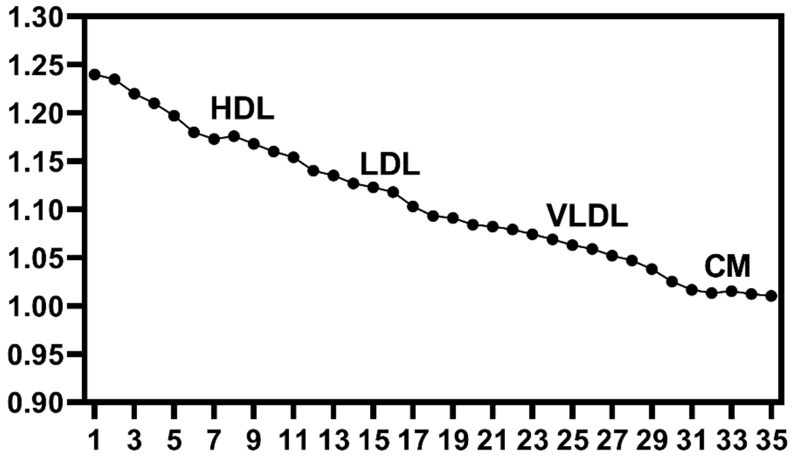

Figure S2. The fraction of density gradient separation for lipoprotein.

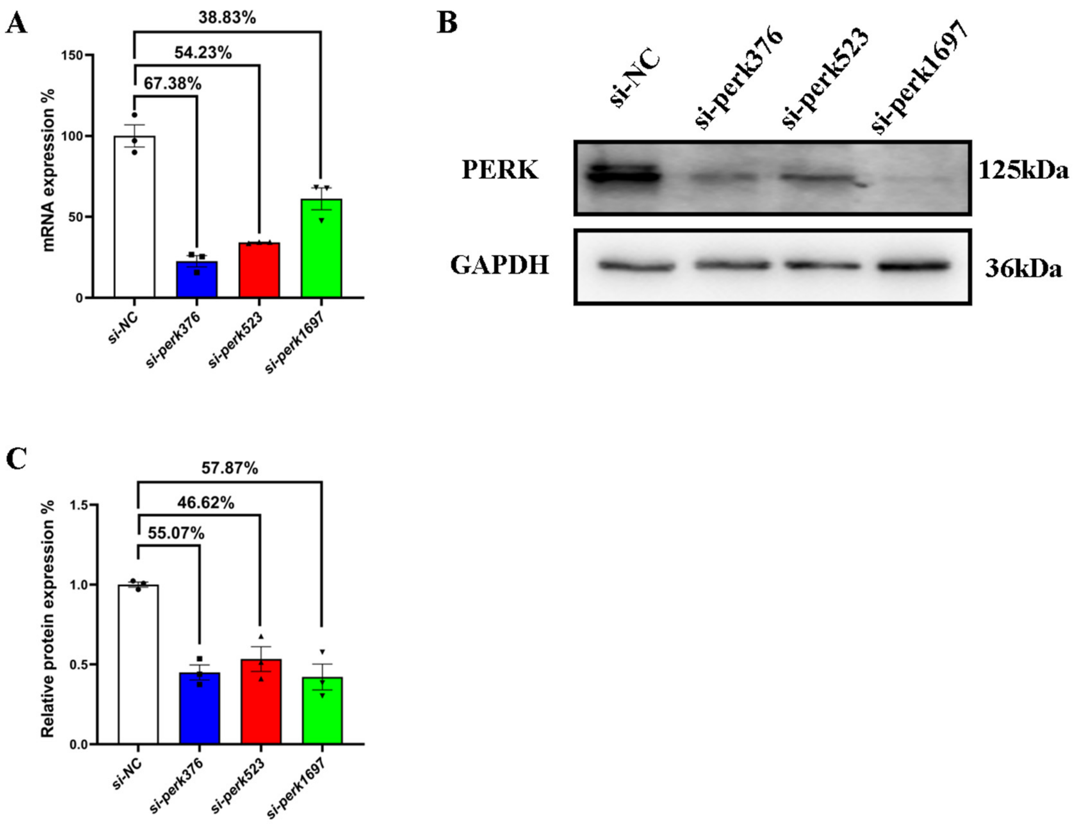

Figure S3. The knockdown efficiency of *perk* gene in IECs. (A) mRNA levels of *perk.* (B-C) Western blot analysis for PERK. Values are means ± SEM, n ≥ 3. Letters denote significance at $P < 0.05$ (One-way ANOVA, Duncan post hoc test).

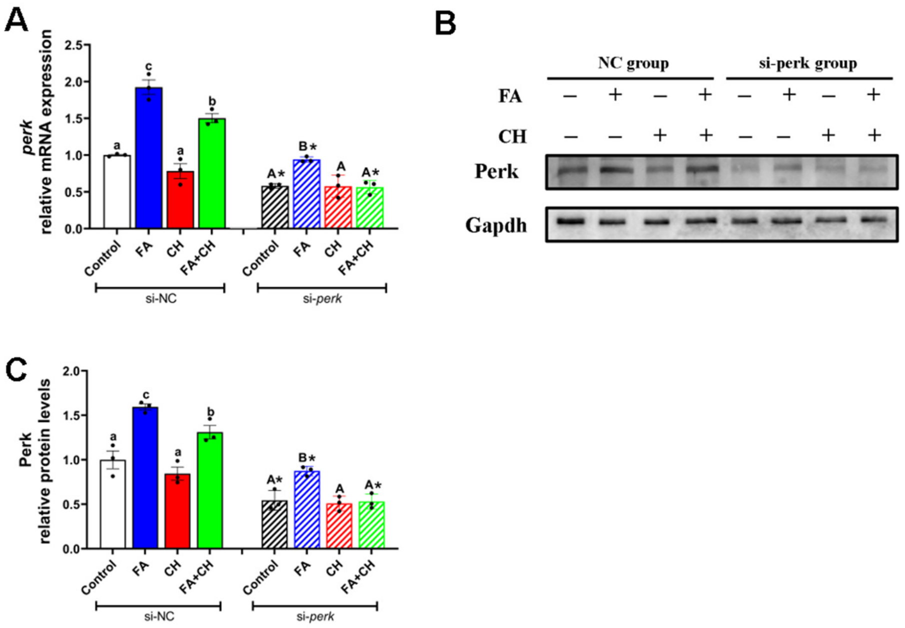

Figure S4. mRNA levels and Western blot analysis of *perk* in the IECs under FA and CH incubation and transfected with si-*perk*. (A) mRNA levels of *perk*. (B-C) Western blot analysis for Perk. Data are mean ± SEM, n = 3 n = 3 independent biological experiments; different minuscules indicate significant differences in si-NC groups, different majuscules indicate significant differences in si-*perk* groups ($p \leq 0.05$); asterisks indicate significant differences between si-NC and si-*perk* groups ($*p \leq 0.05$).

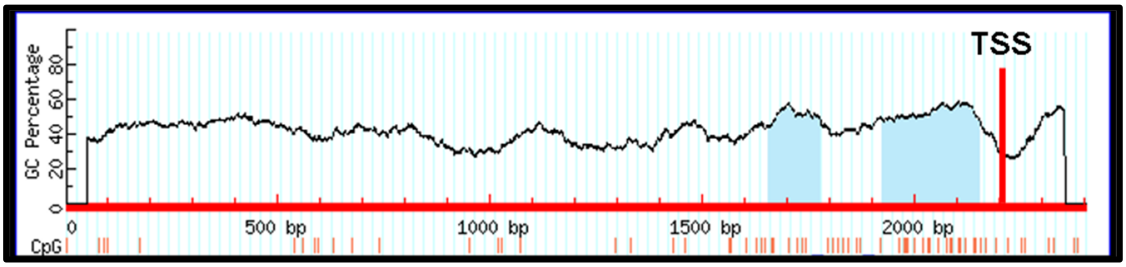

Figure S5. Prediction analysis of CpG islands in the sequence range of 2300 bp upstream from the transcriptional start site in the *perk* promoter region (http://www.urogene.org/). TSS, transcription start site.

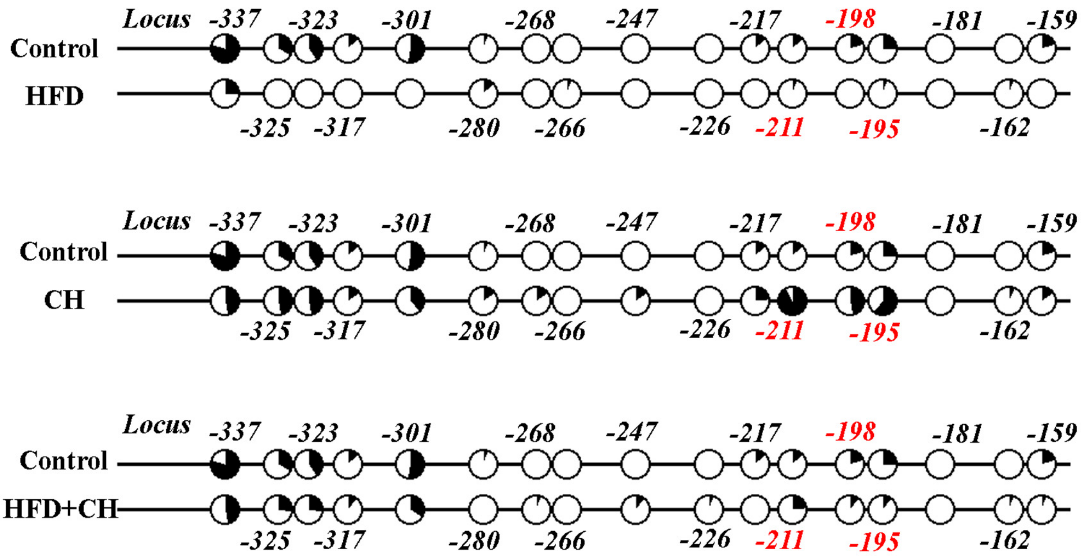

Figure S6. Bisulfite sequencing of the promoters of *perk* between the control, HFD, CH and HFD + CH.

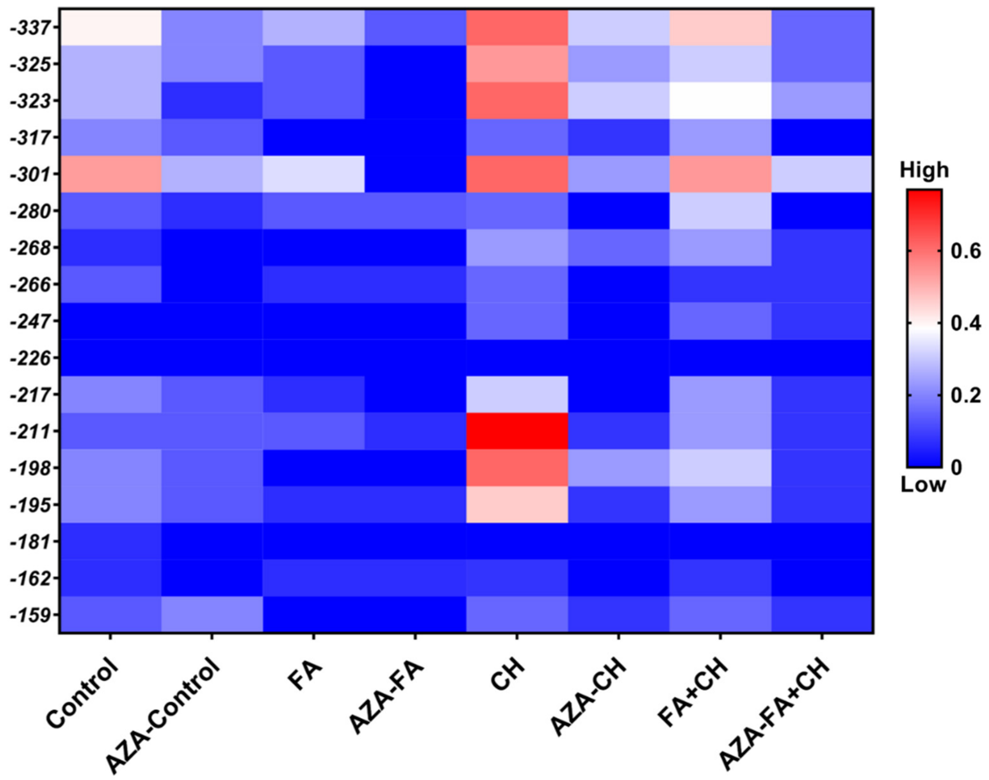

Figure S7. Heat map of methylation percentage of *perk* in the IECs under FA and CH incubation and addition AZA.

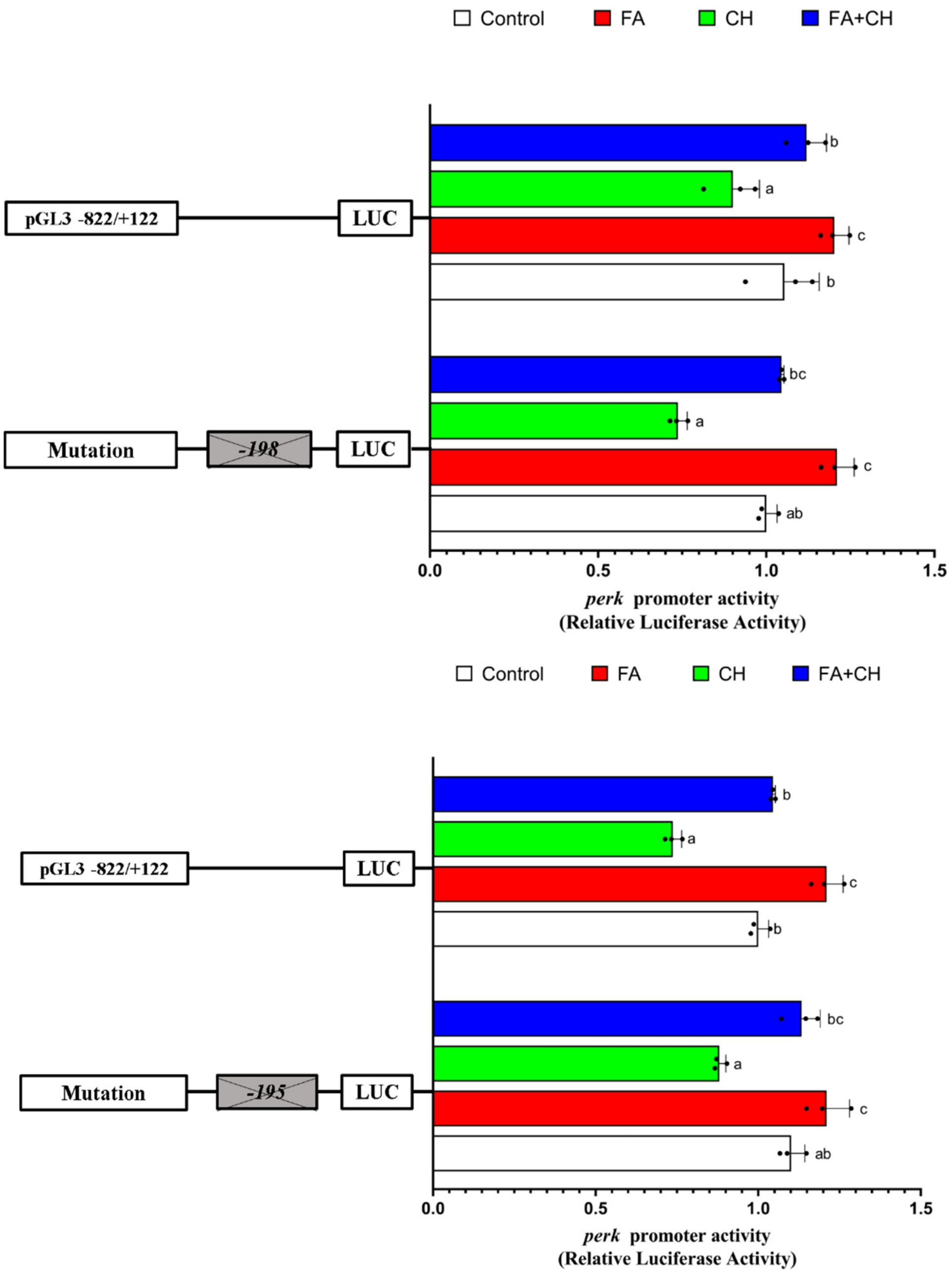

Figure S8. The *-198* and *-195* methylation sites relative luciferase activities of *perk* in the IECs under FA and CH incubation. Values are means ± SEM, n ≥ 3. Letters denote significance at $P < 0.05$ (One-way ANOVA, Duncan post hoc test).
